# An Advanced Nursing Directive for Children With Suspected Appendicitis: Protocol for a Quality Improvement Feasibility Study

**DOI:** 10.2196/33158

**Published:** 2021-10-20

**Authors:** Hanu Chaudhari, Michelle Schneeweiss, Reid Rebinsky, Enrico Rullo, Mohamed Eltorki

**Affiliations:** 1 Faculty of Health Sciences McMaster University Hamilton, ON Canada; 2 Department of Pediatrics McMaster University Hamilton, ON Canada

**Keywords:** quality improvement, pediatric, nursing, medical directive, appendicitis, emergency department flow, nursing directive

## Abstract

**Background:**

Pediatric appendicitis accounts for an estimated 7% to 10% of abdominal pain cases in the emergency department (ED). The diagnosis is time-consuming, and the investigative process depends on physician assessment, resulting in delays in diagnosis and therapeutic management. The utility of an advanced nursing directive (AND) to expedite this process is unclear and needs further exploration.

**Objective:**

This study aims to describe key components of ED flow in patients with suspected appendicitis seen at a pediatric ED and pilot a directive that allows ED nurses to perform an order set that includes blood work, urine tests, analgesics, fluids, and an abdominal-pelvis ultrasound prior to physician assessment.

**Methods:**

This study involves conducting a retrospective chart review alongside a quality improvement initiative to compare key ED flow metrics before and after AND implementation. Primary outcome measures include median time from ED triage assessment to ultrasound completion, analgesia administration, blood work results, and time to disposition (consult or discharge), alongside other key ED flow metrics for suspected appendicitis. Secondary outcomes will involve patient and caretaker satisfaction surveys. Descriptive statistics will be used to summarize the data. For differences in proportions, a chi-square test will be used. The Student *t* test will be used for continuous variables. A variable-controlled run chart will be performed to assess impact on ED flow metrics. Patient and family satisfaction surveys are administered immediately after the directive encounter and 7 days afterward.

**Results:**

There are currently 3900 patients who have been screened, 344 patients who have been enrolled, and 90 patients who have received the medical directive since implementation in June 2020. Interim results on reduction of time to diagnostic and therapeutic ED flow parameters and satisfaction surveys are expected to be published in February 2022. The final study endpoint will be in June 2022.

**Conclusions:**

This study proposes a novel protocol for improving the diagnosis and treatment of suspected pediatric appendicitis through implementation of an evidence-based AND. This model may provide a standardized, international pathway for management of common pediatric and adult emergencies.

**International Registered Report Identifier (IRRID):**

RR1-10.2196/33158

## Introduction

### Background

Acute appendicitis is the most common pediatric surgical emergency [1]. This condition, characterized by inflammation of the appendiceal lumen, accounts for an estimated 7% to 10% of all abdominal pain cases presenting to the emergency department (ED) [2,3]. Although the overall incidence of appendicitis may be declining among Canadian children [4], perforated appendicitis, a serious cause of morbidity and mortality, occurs more frequently in children, making early diagnosis and treatment imperative [5,6].

Appendicitis typically presents with a sequence of acute onset colicky pain to the umbilicus, which then becomes sharp and constant and then migrates to the right lower quadrant (RLQ) [1]. Children frequently present with fever, nausea, vomiting, anorexia, and constipation. Although this classic presentation has been shown to vary depending on the age and dietary patterns (such as decreased fiber intake) of the patient in question [7], the diagnosis is predominantly clinical. The final diagnosis is typically made by ED physicians based on clinical judgment as well as a combination of investigations, including urine analysis, pregnancy tests, complete blood count, inflammatory markers, and ultrasound (US) imaging. Clinical decision rules can also be used to streamline the diagnostic workup but rely on the result of white blood cell count and neutrophils or bands as well as clinical features. In addition to blood work, US is a rate-limiting step in the time to diagnosis of appendicitis, as it is often used for patients who present atypically or who are at intermediate risk based on clinical decision rules. After diagnosis, the surgical team is consulted for definitive surgical or medical management [8]. This entire process is lengthy, with one study demonstrating that the average ED length of stay (LOS) was 464 minutes (7.7 hours), the mean time to analgesia was 252 minutes (4.2 hours), the mean time to US performed was 378 minutes (6.3 hours), and the mean time to appendectomy was 717 minutes (12 hours) in Canadian pediatric hospitals [9].

The utility of an advanced nursing directive (AND) allowing nurses to order blood work and imaging studies, such as US, to expedite the diagnostic process of appendicitis remains unclear. ANDs, also commonly referred to as medical directives, serve to empower nursing staff by enabling them to provide advanced levels of care to patients prior to physician assessment, and they have been shown to reduce ED LOS and time to disposition (discharge, consultation, or admission) [10]. Prior studies have examined nursing-initiated therapeutics, including therapies for asthma [11,12], pulled elbow reductions [13], radiograph ordering for suspected fractures [14], and oral rehydration pathways for gastroenteritis [15]. One meta-analysis of four studies investigating a clinical decision rule that allowed nurses to order ankle radiographs showed that there were significantly fewer x-rays (odds ratio 0.36, 95% CI 0.22-0.59) with no difference in proportions of positive ankle fracture x-rays or missed fractures, as well as a 35-minute reduction in ED LOS when comparing the triage nurses using this clinical decision rule to physicians [16]. For therapeutic interventions, ANDs have been shown to reduce the time to analgesia by an average of 30 minutes, which resulted in significant reductions in pain scores and increases in patient satisfaction rates [17].

Initial research has shown the strong potential of ANDs to expedite and improve the quality of patient care in the ED without increasing ED resource use for various conditions. However, there is a lack of research exploring the utility of an AND for the workup of children with suspected appendicitis. Thompson et al [18] have shown that ANDs that empower nurses to begin investigations prior to physician assessment have resulted in a significant reduction in time of triage to blood draw, hospital admission, and surgical appendectomy. However, the AND used in this study did not allow nurses to order imaging studies in cases of suspected appendicitis, resulting in no reported difference in time to US between groups. As this is a key investigation in confirming the diagnosis, it is essential to determine if expediting time to US can also improve patient outcomes and ED flow metrics.

### Aims and Objectives

To build on the early work by Thompson et al [18], we have designed a novel AND that allows nurses in our ED to order imaging studies in patients with suspected appendicitis. Our primary goal with the implementation of this novel AND is to reduce ED LOS and time to disposition for patients presenting with suspected appendicitis by 20% from baseline. Our secondary goals are to decrease time to other key steps in the diagnostic and therapeutic management of patients with suspected appendicitis, including times to initiating blood work, fluid filling of bladder, and analgesia. Moreover, through the implementation of this AND, we aim to improve the satisfaction levels of both patients with suspected appendicitis and caregivers when presenting to the ED.

## Methods

### Study Design

The implementation of this novel AND was designed as a quality improvement (QI) initiative. Data will be collected to compare the outcomes of a standard of care (SOC) group against a group of patients that received the AND. The protocol for this study was approved by the Hamilton Integrated Research Ethics Board.

### Development of the AND

This novel AND was designed in collaboration with physicians, allied health care workers, members of our institution’s family council, and hospital management leaders. This project was developed to build on a prior successful appendicitis QI project from our institution [19], which was a clinical pathway that assisted physicians in risk stratification of patients with suspected appendicitis after US completion to expedite disposition.

We reviewed previously validated appendicitis scoring systems and, via group consensus, chose to base our directive off the pediatric appendicitis score (PAS) [20]. This screening tool is simple, and when used in a clinical pathway that includes advanced imaging such as US, there is a high sensitivity and specificity (92% and 95%, respectively), a positive likelihood ratio of 17.3, and a negative likelihood ratio of 0.08 [21]. Our screening tool made slight modifications to the original PAS because nursing assessment at our institution is done prior to laboratory investigations. Criteria such as complete blood count results were not included in our AND (Table 1). According to the PAS, a score of ≥4 is medium risk and will require investigation for appendicitis using laboratory work and US. Patients with RLQ tenderness to cough, percussion, or hopping alongside tenderness on palpation over the RLQ are given a score of 4 (medium risk) on the PAS. Therefore, appendicitis cannot be ruled out without further investigation. For our AND, we included patients who present with abdominal pain and have the above two clinical features.

During the conception of this study, our team wanted to ensure we appropriately addressed the interest and viability of the methods with nursing staff. We sought the input of several nurses and our unit’s educational nurse when designing the AND. We surveyed all the nurses in our ED to assess their perceptions of the AND. A total of 52 nurses (full and part-time) were invited to complete the nursing survey regarding their perceptions of the AND. Of the 52 nurses, 39 completed our survey (75% response rate). In total, 85% (33/39) of all nurses were comfortable assessing the abdomen for RLQ pain. The respondents estimated that 85% of the time, there is agreement between nurses and physicians regarding whether a possible appendicitis diagnosis requires further investigation. In addition, 90% (35/39) of respondents thought that the AND would improve patient flow metrics (time to disposition, ED LOS), improve patients’ experience, and empower nurses to facilitate patient care.

**Table 1:** Criteria for the original PAS versus the AND-modified PAS.

Signs and symptoms	PAS^a^ criteria	AND^b^-modified PAS criteria for screening eligible patients
RLQ^c^ tenderness to cough, percussion, or hopping	No=0 Yes=+2	No=0 Yes=+2
Anorexia	No=0 Yes=+1	N/A^d^
Fever (temperature ≥38 ºC)	No=0 Yes=+1	N/A
Nausea or vomiting	No=0 Yes=+1	N/A
Tenderness over right iliac fossa	No=0 Yes=+2	No=0 Yes=+2
Leukocytosis (WBC^e^ >10,000)	No=0 Yes=+1	N/A
Neutrophilia (ANC^f^ >7500)	No=0 Yes=+1	N/A
Migration of pain to RLQ	No=0 Yes=+1	N/A

^a^PAS: pediatric appendicitis score.

^b^AND: advanced nursing directive.

^c^RLQ: right lower quadrant.

^d^N/A: not applicable.

^e^WBC: white blood cell.

^f^ANC: absolute neutrophil count.

### Eligibility Criteria, Setting, and Sampling

This novel AND will be implemented at our institution that serves a catchment area of 2.3 million people with an annual ED volume of approximately 50,000 patients. After consultation with our institutions’ nurses, nurse educator, and family council, we determined that the AND should be applied to children with classic appendicitis symptoms that have minimal comorbidities. The patient eligibility criteria listed in Textbox 1 are required by the bedside nurse to initiate care as directed by the AND.

**Eligibility criteria for patients with suspected appendicitis to receive the AND.**

**Inclusion criteria**

- Age 3–17 years (children ≤2 years of age are at low risk for appendicitis and present atypically [7])
- RLQ abdominal pain with cough, jump, or percussion
- Right iliac fossa tenderness
- Symptoms ≤4 days in duration (longer duration of pain is less likely to be appendicitis [7])

**Exclusion criteria**

- Prior abdominal surgery, excluding extraperitoneal (eg, inguinal hernia repair)
- Implantable abdominal devices (eg, shunt, dialysis, catheter)
- Any of the following comorbid conditions: diabetes, immunocompromised, sickle cell disease, active rheumatological conditions, active cancer, inflammatory bowel disease, short gut or Hirschsprung disease, or 24-hour trauma diagnosis

### Directive Implementation

**Figure 1:**
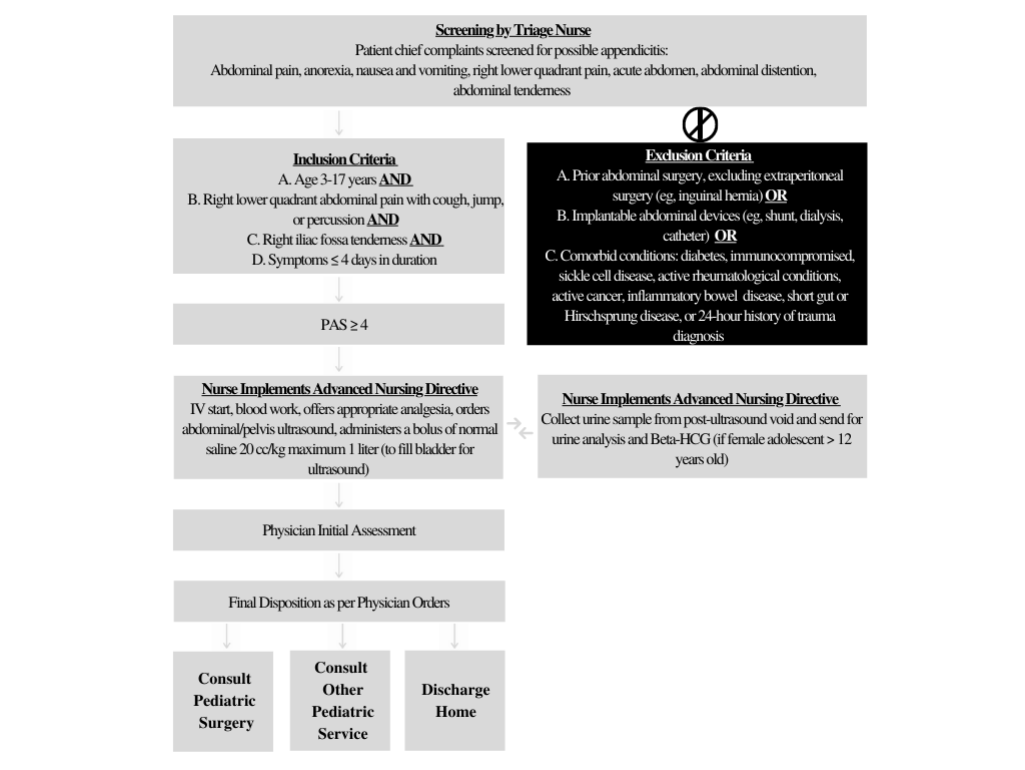
Advanced nursing directive algorithm for children with suspected appendicitis. HCG: human chorionic gonadotropin; IV: intravenous; PAS: pediatric appendicitis score.

There are two simultaneous phases to the directive implementation. During phase 1, we ran educational sessions for all nurses and child life specialists on signs and symptoms of appendicitis for screening, and on the components of the PAS and the AND. In the post-implementation period (phase 2), triage nurses screen children who present with chief concerns that are related to appendicitis (abdominal pain, RLQ pain, vomiting, anorexia, acute abdomen, abdominal distension, and abdominal tenderness). If the child meets the inclusion criteria for suspected appendicitis, the patient’s chart will be flagged, and they will be prioritized in a room. However, they are to remain in the same order to be seen by the physician, which is determined by the Canadian Triage and Acuity Scale (CTAS) score and time of arrival, so as not to impact the flow of other patients through the ED. Once in a room, the primary nurse completes primary assessment, the AND, and a PAS. For patients with a score of ≥4, an emergency nurse can perform the procedures shown in Figure 1 and Textbox 2 before assessment by an ED physician.

**Advanced nursing directive order set.**

- Establish intravenous access
- Obtain blood work (complete blood count/differential, electrolyte levels, C-reactive protein)
- Order diagnostic imaging, including abdominal/pelvic ultrasound
- Administer a bolus of 0.9% normal saline at 20 cc/kg, with a maximum of 1 liter to fill bladder for ultrasound (a requirement in our center to displace bowel out of pelvis)
- Offer analgesia consisting of intravenous ketorolac for moderate to severe pain or oral ibuprofen/acetaminophen for mild pain and document whether analgesia was received or declined
- Collect urine from the post-ultrasound void and send for routine urine analysis; send for urine culture if urinalysis is positive for nitrites or leukocytes and order point-of-care pregnancy test (beta-human chorionic gonadotropin) for female adolescent patients (>12 years old)

### Patient and Caretaker Satisfaction Survey

We will administer a patient satisfaction survey before and after the implementation of the AND. The survey was adapted from the Emergency Department Patient Experience of Care (EDPEC) survey developed in the United States [22], which is a standardized, valid, and reliable questionnaire to measure adult patients’ experience of ED care (see Multimedia Appendices 1 and 2).

### Outcome Measures

Primary outcome measures include time to (measured from triage, in minutes) (1) intravenous (IV) catheter insertion, (2) blood work results, (3) analgesia administration, (4) IV fluid completion, (5) US requisition fax time, (6) US completion, (7) US reporting, (8) disposition (time to consult or discharge), and (9) ED LOS. Secondary outcome measures include (1) patient satisfaction (as measured on patient satisfaction surveys) and (2) balancing measures such as the proportions of laboratory tests, urine tests, and US ordered for possible appendicitis before and after the implementation of AND.

### Data Collection

To collect data from both the SOC group and the AND group, we will retrospectively screen the charts of patients who meet the inclusion and exclusion criteria outlined above. To do this, we queried the decision support team at our institution for all pediatric ED visits between April 2018 and June 2020 stratified based on the chief concerns as shown in Figure 1.

A standardized case report form will be used for data collection, including demographics (ie, sex and age), symptoms at the onset of presentation, results of relevant laboratory investigations (ie, complete blood count, electrolytes, beta-human chorionic gonadotropin), relevant imaging results (ie, US results), disposition (ie, home, admission to hospital), treatment modalities (ie, antibiotics, pain medications, surgery), and time to each of the steps in this workup (in minutes) (see Multimedia Appendix 3).

No personal identifiers, such as the patient’s name, will be collected or recorded on the study forms. Instead, each participant will be given an enrollment number. Patients from the SOC group will be assigned a screening number beginning with MCH-S and a unique 4-digit code (eg, MCH-S-1234); those who are eligible based on the inclusion and exclusion criteria will receive an enrollment number beginning with the letters MCH-E and ending with a unique 4-digit code (eg, MCH-E-1234). Patients from the AND group will be given an enrollment number beginning with the letters MCH-D and ending with a unique 4-digit code (eg, MCH-D-1234). Two designated research members will then review a proportion of the case report forms at random for completion and discrepancies. Incomplete or discrepant data will be ameliorated by a third independent reviewer.

### Data Entry

All study data will be entered into an electronic database, Research Electronic Data Capture (REDCap), by study team members at our institution. The REDCap database will be maintained and accessible only within our institution. All study data will be identified by unique study IDs only, as previously mentioned.

### Statistical Analysis

Patient characteristics will be summarized overall and by phase of study to compare before and after implementation of AND. Categorical variables will be summarized using frequencies, proportions, and rates. Continuous variables will be summarized using means, medians, SDs, and IQRs where appropriate. For patient and provider baseline characteristics, chi-square and Fisher exact tests will be used to compare categorical variables, and the Student *t* test or Wilcoxon rank sum test will be used for continuous variables between two phases of the study. Baseline characteristics of patients in the two phases of the study will be tested. In the time-to-event analysis (time to disposition), we will censor for higher acuity patients (CTAS 1 and 2) because they are usually prioritized for physicians to see them quickly. The QI statistical process control run chart will be used to detect trends or patterns over the study time to demonstrate sustained change. SAS 9.2 (SAS Institute Inc) will be used for all analyses.

## Results

The project was funded in June 2019 and approved by the research ethics board in February 2020. As of August 2021, for the retrospective SOC group, 3900 patients had been screened and 344 patients had been enrolled. There are currently 90 patients who have received the medical directive since its implementation in June 2020. The final study endpoint will be in June 2022. Interim results on reduction of time to diagnostic and therapeutic ED flow parameters and patient satisfaction are expected to be published in February 2022.

## Discussion

### Projected Significance

The projected significance of this study is to improve clinical outcomes for pediatric patients, empower nurses, increase patient and family satisfaction, reduce ED overcrowding, and improve ED flow metrics. ED wait times in Canada have been shown to lead to increased mortality and morbidity [23]; yet, there has been limited action to develop sustainable strategies to address this. COVID-19 caused a significant upheaval in ED capacity and volumes [24], and as the looming threat of variants continues, it is important to ensure that patients, especially those presenting with potentially emergent conditions, are seen more efficiently. Our study provides a novel method of addressing these concerns in a framework that can be applied to many other emergent clinical diagnostic pathways beyond appendicitis (eg, testicular torsion) and in both academic and community hospital sites.

### Limitations

We aim to address several limitations in this study. First, this directive is heavily reliant on patient volumes, physician/nursing staffing, and time of patient arrival to the ED, as these directly affect ED flow metrics. To address this, an ED run chart will be constructed to visualize the impact the directive has had on primary outcomes and will be adjusted for these variables. To do this, we will record ED flow metrics for both patients who did and did not receive the directive throughout the study period. Second, it is necessary that this directive improves clinical outcomes without increasing resource use. This is especially important in ensuring that the results are better not simply because more imaging and blood work is being ordered but because they are being done for appropriate patient indications/presentations. This potential limitation will be addressed by analyzing the difference of proportion of patients who had a surgical appendectomy that were investigated using the nursing directive versus those that were physician initiated. Finally, although the directive may improve time to initial diagnostic imaging or to disposition (consult or discharge), it may not influence the speed at which teams such as radiology or pediatric surgery can perform imaging, assess the patient, and perform an appendectomy. Given the interprofessional nature of the project, the goal will be to iterate and gain feedback from other departments on how best to reduce these potential bottlenecks in our interim analysis and initial pilot results. In terms of patient/caretaker satisfaction, many factors affect a patient’s experience beyond the ED, which may bias these results. As caretakers and patients can find it difficult to delineate the care in the ED from the ED staff versus surgery, pediatrics, or other providers once transferred, it will be important to keep this in mind when evaluating qualitative feedback.

### Comparison With Prior Work

Two prior studies [18,19] with similar concepts served as the basis for this integrative study. The authors involved with the studies have also served as collaborators for this study (GT, HF). The study done by Thompson et al [18], as previously mentioned, showed a significant time to reduction in time of triage to blood draw, hospital admission, and appendectomy. Our study adds additional outcomes such as patient satisfaction and time to US to further understand the effectiveness of this directive in quality of care and diagnostic efficiency. Another study [19] that was conducted at our institution examined the implementation of a standardized appendicitis care pathway for ED physicians. This study found that this process could reduce negative appendectomies, unnecessary computed tomography scans, and unnecessary hospital admissions. Our study built on this research and implemented this into the directive pathway, especially as it pertains to US imaging; however, we focused on nursing staff instead of physicians, as they are the first to see the patient prior to initial assessment by the physician.

### Conclusions

Pediatric appendicitis is a common surgical emergency that can be diagnosed and treated more efficiently using an evidence-based advanced nursing medical directive. This initiative can improve patients’ therapeutic outcomes, quicken diagnostic outcomes, empower nurses to begin the diagnostic workup, and improve patient and caretaker satisfaction with treatment provided in the ED. Our future goals are to publish the results of this initial pilot study and begin working with collaborators to implement this initiative into other institutions.

## Appendix

Multimedia Appendix 1 Patient and caretaker satisfaction survey completed after the initial directive encounter.

Multimedia Appendix 2 Patient and caretaker satisfaction survey completed 7 days after the initial directive encounter.

Multimedia Appendix 3 Case report form for data capture on standard of care and directive patients.

Multimedia Appendix 4 Funded peer review letter.

## Abbreviations

AND: advanced nursing directive
CTAS: Canadian Triage and Acuity Scale
ED: emergency department
IV: intravenous
LOS: length of stay
PAS: pediatric appendicitis score
QI: quality improvement
REDCap: Research Electronic Data Capture
RLQ: right lower quadrant
SOC: standard of care
US: ultrasound
