# Supplementary material for: An Advanced Nursing Directive for Children With Suspected Appendicitis: Protocol for a Quality Improvement Feasibility Study
Source: JMIR Res Protoc. 2021 Oct 20;10(10):e33158. doi: 10.2196/33158 (PMC8567150; doi:10.2196/33158)
Supplement: Multimedia Appendix 1 [file resprot_v10i10e33158_app1.docx]

Pre-Implementation Patient Experience of Care Survey

Advanced Nursing Directive for Appendicitis

*(McMaster Children’s Hospital Emergency Department)*

SURVEY INSTRUCTIONS

Answer all the questions by checking the box to the left of your answer.

You are sometimes told to skip over some questions in this survey. When this happens you will see an arrow with a note that tells you what question to answer next, like this:

Yes

No→ *If No, Go to Question 1*

All of the questions in the survey will ask about this emergency department visit.

*Questions 1 to 31 and 34-36 were adapted from the* [*Emergency Department Patient Experience of Care (EDPEC)*](https://www.cms.gov/Research-Statistics-Data-and-Systems/Research/CAHPS/ed.html) *survey developed in the United States.*

1. Who is completing the survey?

□ Mother

□ Father

□ Grandmother

□ Grandfather

□ Aunt

□ Uncle

□ Yourself

□ Other: _______________________________________________________

1. In the last 6 months, how many times have you visited any emergency department to get care for yourself? (does not include this ED visit).

□ 1 time

□ 2 times

□ 3 times

□ 4 times

□ 5 to 9 times

□ 10 or more times

1. When you first arrived at the emergency department, how important was it for you to get care right away by an ED registered nurse?

Extremely important

Extremely not important

1. How important was it to be seen right away by a physician during this Emergency Department visit?

Extremely not important

Extremely important

1. How satisfied were you with the wait time to see a nurse?

Extremely dissatisfied

Extremely satisfied

1. How satisfied were you with the wait time to see a physician?

Extremely satisfied

Extremely dissatisfied

1. During this emergency department visit, did you (or your child) have any pain (prior to receiving any pain medication?

For age <6 years old:


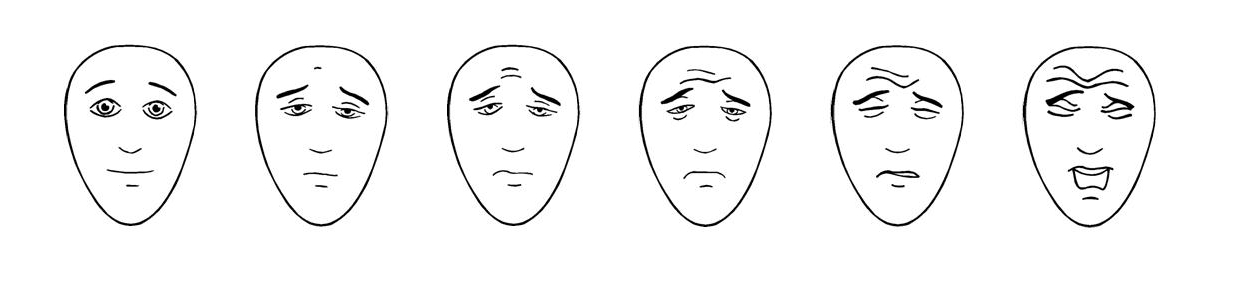
For age ≥ 6 years old


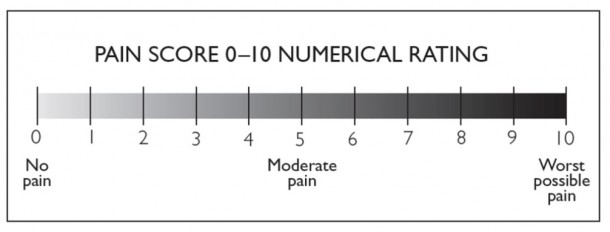


1. During this emergency department visit, did you receive any pain medications?

□ Yes

□ No (Proceed to question 11)

1. How comfortable were you with a nurse starting you on pain medications prior to the physician assessing or seeing you?

Extremely Un-comfortable

Extremely comfortable

1. How satisfied were you with the wait time for receiving pain medication?

Extremely dissatisfied

Extremely satisfied

1. If you had an ultrasound, how satisfied were you with the wait time for imaging studies (e.g. ultrasound) for you?

Extremely satisfied

Extremely dissatisfied

□ No ultrasound

1. How comfortable were you with a nurse ordering an ultrasound for you prior to the physician assessing or seeing you?

Extremely comfortable

Extremely Un-comfortable

1. If you had bloodwork, how satisfied were you with the wait time for bloodwork to be drawn for you (or your child)?

Extremely satisfied

Extremely dissatisfied

□ No blood work

1. How comfortable were you with a nurse performing blood work prior to the physician assessing or seeing you?

Extremely Un-comfortable

Extremely comfortable

1. During this emergency department visit, how often did nurses listen carefully to you?

| 5  Always | 4  Often | 3  Sometimes | 2  Rarely | 1  Never |
| --- | --- | --- | --- | --- |

1. Is there a specific diagnosis or problem that you were suspicious of based on the symptoms (i.e. what were you worried about?) that made you come to the Emergency Department?

□ Appendicitis

□ Ovarian cyst

□ Urine infection

□ Stomach flu

□ Hernia

□ Pneumonia

□ Other:

_____________________________________________________________________

1. What was your final diagnosis during this Emergency Department Visit?

- Appendicitis
- mesenteric adenitis
- gastroenteritis
- constipation
- pneumonia
- UTI
- renal calculi (kidney stones)
- ovarian cysts
- ovarian torsion
- testicular torsion
- hepatobiliary calculi
- abdominal pain without a specific diagnosis
- abdominal tumor
- Other:

1. During this emergency department visit, did doctors spend enough time with you?

| 5  Yes definitely | 4  Yes probably | 3  Neutral | 2  Probably not | 1  Definitely not |
| --- | --- | --- | --- | --- |

OVERALL EXPERIENCE

Please answer the following questions about your visit to the emergency department named on the front of the survey. Do not include any other emergency department visits in your answers.

1. Using any number from 0 to 10, where 0 is the worst care possible and 10 is the best care possible, what number would you use to rate your care during this emergency department visit?

Worst care possible

po

Best care possible

1. Would you recommend this emergency department to your friends and family?

| 5  Yes definitely | 4  Yes probably | 3  Neutral | 2  Probably not | 1  Definitely not |
| --- | --- | --- | --- | --- |

1. Overall, how long did your visit to the emergency department last?

□ Less than 1 hour

□ 1-3 hours

□ 4-6 hours

□ 7-9 hours

□ 10-12 hours

□ > 12 hours

1. Overall, during this emergency department visit, did you have to wait too long to get care?

| 5  Yes definitely | 4  Yes probably | 3  Neutral | 2  Probably not | 1  Definitely not |
| --- | --- | --- | --- | --- |

1. Is there anything else you would like to share about your Emergency Department visit?

Thank you.

Please return the completed survey in the postage-paid envelope.
