# Supplementary material for: An Advanced Nursing Directive for Children With Suspected Appendicitis: Protocol for a Quality Improvement Feasibility Study
Source: JMIR Res Protoc. 2021 Oct 20;10(10):e33158. doi: 10.2196/33158 (PMC8567150; doi:10.2196/33158)
Supplement: Multimedia Appendix 2 [file resprot_v10i10e33158_app2.docx]

**Appendicitis Advanced Nursing Directive**

**Post-ED Visit Telephone Survey**

1. What was the diagnosis given at the end of your first ED visit or hospital stay?

- Appendicitis
- mesenteric adenitis
- gastroenteritis
- constipation
- pneumonia
- UTI
- renal calculi (kidney stones)
- ovarian cysts
- ovarian torsion
- testicular torsion
- hepatobiliary calculi
- abdominal pain without a specific diagnosis
- abdominal tumor
- Other:

1. Did you make any additional visits to an ED or an urgent care within 7 days of your visit at our ED?

- Yes (Go to Question 3)
- No (Go to Question 7)

1. Which ED or urgent care did you visit?

- MUMC
- Other ED (e.g. WLMH, St Joes, HGH, St Catherine EDs)
- Other Urgent Care (e.g. main street west urgent care)

1. Did you receive any alternative diagnoses in this subsequent Emergency Department visit?

- Yes
- No

1. Were there any additional investigations completed?

- Blood work
- abdominal Xray
- Chest Xray
- Ultrasound
- CT
- Urine tests
- Other: _____________________

1. What was the alternative diagnosis?

- Appendicitis
- mesenteric adenitis
- gastro
- constipation
- pneumonia
- UTI
- renal calculi (kidney stones)
- ovarian cysts
- ovarian torsion
- testicular torsion
- hepatobiliary calculi
- abdominal pain without a specific diagnosis
- abdominal tumor
- Other:____________________________________________________

1. Have you seen your family physician for follow up within 7 days from the ED visit?

- Yes
- No (skip to question 10)

1. Did you receive any alternative diagnoses by your family physician other than your ED discharge diagnosis?

- Yes
- No (skip to question 10)

1. What was the alternative diagnosis?

- Appendicitis
- mesenteric adenitis
- gastro
- constipation
- pneumonia
- UTI
- renal calculi (kidney stones)
- ovarian cysts
- ovarian torsion
- testicular torsion
- hepatobiliary calculi
- abdominal pain without a specific diagnosis
- abdominal tumor
- Other:____________________________________________________

1. Have you seen any other health care provider outside of an ED, urgent care, or family medicine?

- Yes
- No (go to question 12)

1. Which specialty did you see?

- Pediatric Surgery
- Pediatrics
- Urology
- GI
- Other:

1. Did the diagnosis change?

- Yes
- No (go to question 14)

1. What was the alternative diagnosis?

- Appendicitis
- mesenteric adenitis
- gastro
- constipation
- pneumonia
- UTI
- renal calculi (kidney stones)
- ovarian cysts
- ovarian torsion
- testicular torsion
- hepatobiliary calculi
- abdominal pain without a specific diagnosis
- abdominal tumor
- Other:____________________________________________________

1. Did any adult caregiver to the child work schedule get affected by their child’s illness?

- Yes
- No (skip to question 16)

1. How many days of work did the caregiver miss as a result of this Emergency Department visit? (if had multiple visits, remind patient/family with their specific date of the first visit to the ED)

- No missed days
- 1 day
- 2 day
- 3 day
- 4 day
- ≥ 5 days
- Other: _______________________

1. How many days of school or day care did your child miss as a result of this Emergency Department visit?

- Does not go to school or day care
- No missed days
- 1 day
- 2 day
- 3 day
- 4 day
- ≥ 5 days
- Other: _______________________

1. From 1-10 (1 extremely dissatisfied, 10 extremely satisfied), how would you rate your overall experience in the emergency department?

- 1
- 2
- 3
- 4
- 5
- 6
- 7
- 8
- 9
- 10

1. From 1-10 (1 extremely dissatisfied, 10 extremely satisfied), how would you rate your overall experience with the investigations you or your child received for their condition?

- 1
- 2
- 3
- 4
- 5
- 6
- 7
- 8
- 9
- 10

1. From 1-10 (1 extremely dissatisfied, 10 extremely satisfied), how satisfied are you with the diagnosis you or your child received for their condition?

- 1
- 2
- 3
- 4
- 5
- 6
- 7
- 8
- 9
- 10
