# Supplementary material for: An Advanced Nursing Directive for Children With Suspected Appendicitis: Protocol for a Quality Improvement Feasibility Study
Source: JMIR Res Protoc. 2021 Oct 20;10(10):e33158. doi: 10.2196/33158 (PMC8567150; doi:10.2196/33158)
Supplement: Multimedia Appendix 3 [file resprot_v10i10e33158_app3.docx]

| **CRF- Quality Improvement Appendicitis Chart Review** |
| --- |

**Study ID** (from the Screening Form):

| M | C | H | -- | E | -- |  |  |  |
| --- | --- | --- | --- | --- | --- | --- | --- | --- |

Site Number Form Patient Number

**IMPORTANT NOTE:**

When assigning Study ID, it is VERY IMPORTANT to ensure the participant is eligible. DO NOT assign Study ID if patient is not eligible.

Screening ID :

| M | C | H | -- | S | -- |  |  |  |
| --- | --- | --- | --- | --- | --- | --- | --- | --- |

Site Form Participant Number

**Date and Time of Emergency Presentation:** _______/_______/_________   _____:_____

**YYYY / MM / DD hh : mm**

**Age of the child (years and fraction of years, e.g. 5 years 10 months=5.8)** _______ years.

**Meet Eligibility criteria (Meets all inclusion criteria and No exclusion criteria)** : □ Yes □ No

**NOTE: IF THE PARTICIPANT IS NOT ELIGIBLE, DO NOT PROCEED ANY FURTHER.**

**CHART REVIEW**

**Triage Weight:** _______ Kgs □ Not Charted

**Gender:** □ Male □ Female □ Other ______

**Triage time:** _________

**Time to ED physician initial assessment** ____________ □ Not charted

(*usually written by MD top of chart beside physician notes, hh:mm in 24 hour format e.g. 1830*)

**TRIAGE Vitals:**

Temperature ____ C Heart Rate ____ Blood Pressure: ____/____ Respiratory Rate____

**CTAS:** 1 □ 2 □ 3 □ 4 □ 5 □

**CHIEF COMPLAINT**

Abdominal pain □ Yes □ No □ Not charted

Right lower quadrant abdominal pain □ Yes □ No □ Not charted

Possible appendicitis □ Yes □ No □ Not charted

Vomiting □ Yes □ No □ Not charted

Other __________

**HISTORY OF PRESENTING ILLNESS**

**Duration of symptoms** (general onset of illness, in days and fractions of days, e.g. if symptoms started 6 hours ago, answer should be 0.25 days): ___________ days

**Abdominal pain** □ Yes □ No □ Not charted

If yes:

Onset in days (or fraction of days): __________ days □ Not charted

Migration to Right lower quadrant □ Yes □ No □ Not charted

(started around belly button then moved down)

Pain with walking/jumping/movement (e.g. pain during car ride, or bed shaking) □ Yes □ No □ Not charted

Location of abdominal pain (ON HISTORY) (click all that applies)

- - Periumbilical abdominal pain (around belly button) □
  - Right lower quadrant pain (right iliac fossa) □
  - Left lower quadrant □
  - Right upper quadrant □
  - Left upper quadrant □
  - Suprapubic □
  - Flank pain □
  - Scrotal □

**Vomiting** □ Yes □ No □ Not charted

Number of episodes in the last 24 hours: __________

Onset (days and fraction of days allowed): __________ □ Not charted

**Nausea** □ Yes □ No □ Not charted

**Anorexia or decrease in appetite** □ Yes □ No □ Not charted

Onset (days and fraction of days allowed): __________ □ Not charted

**Abdominal distention** □ Yes □ No □ Not charted

**Fever** □ Yes □ No □ Not charted

Onset (days and fraction of days allowed) ____ □ Not charted

Home measurement _________ °C □ Not charted

Tactile Fever □ Yes □ No □ Not charted

**Diarrhea** □ Yes □ No □ Not charted

Onset (days and fraction of days allowed) ____

Number of episodes in the last 24 hours ____ □ Not charted

**Constipation** □ Yes □ No □ Not charted

*(hard bowel movements, straining, infrequent bowel movements or NO bowel movement)*

Onset (days and fraction of days allowed) ____

**Last bowel movement** *(in days and fraction of days)* ________ □ Not charted

**Blood in stool** □ Yes □ No □ Not charted

**Dysuria** □ Yes □ No □ Not charted

Onset ____ □ Not charted

**Hematuria** □ Yes □ No □ Not charted

Onset____ □ Not charted

**Flank pain** □ Yes □ No □ Not charted

Onset ____ □ Not charted

Radiation (e.g. groin, right lower abdomen):____________ □ Not charted

***Associated Symptom History:***

**Vaginal Bleeding/Discharge** □ Yes □ No □ Not charted

Onset *(in days and fraction of days)*: ____________

**Pregnancy** □ Yes □ No □ Not charted

**Previous STI** □ Yes □ No □ Not charted

**Surgical History:** □ Yes □ No □ Not charted

Type of surgery____

**Medications**: □ Yes □ No □ Not charted

List (name of medication, dose and frequency, e.g. amoxicillin, 200 mg Q8H for 10 days)

_________________________________________________________________________

__________________________________________________________________________________________________________________________________________________

**CLINICAL EXAM**

**(found under physical examination designated spot on chart, also look for text written in the main body, only include the initial physical examination and not any reassessments):**

**General Appearance** □ Well (e.g. well, playing, no distress)

□ ill (lethargic, tired appearing, crying, in distress, dry appearing, toxic)

□ Not charted

**Abdominal distension** □ Yes □ No □ Not charted

**Generalized Abdominal tenderness** □ Yes □ No □ Not charted

**Localized abdominal tenderness or guarding on exam:** □ Yes □ No □ Not charted

CHECK ALL LOCALIZED TENDER SPOTS DOCUMENTED:

e.g. sometimes illustrated on chart: this example is for right iliac fossa tenderness.

Periumbilical □

Epigastric □

Suprapubic □

Right iliac fossa (or abdominal) □


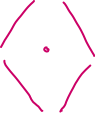


Left iliac fossa (or abdominal) □

Right upper quadrant □


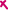


Left upper quadrant □

Right flank or costovertebral angle □

Left flank or costovertebral angle □

**Rebound tenderness** □ Yes □ No □ Not charted

Psoas Sign □ Yes □ No □ Not charted

Also known as obturator sign or pain in the lower quadrant with hyperextension lower limb.

Roving Sign □ Yes □ No □ Not charted

positive when pressure over the patient's *left* lower quadrant causes pain in the right lower quadrant. This sign is sometimes called indirect tenderness.

**Involuntary guarding**  □ Yes □ No □ Not charted

(Abdominal muscle rigidity that the patient cannot relax with palpation)

**Voluntary guarding** □ Yes □ No □ Not charted

(Patient voluntarily tightens abdominal muscles during

palpation by the physician, but can relax with distraction)

**Bowel sounds** □ Yes □ No □ Not charted

**Organomegaly** □ Yes □ No □ Not charted

If yes,

Splenomegaly □

Hepatomegaly □

Mass □

**Hernia exam** □ Yes □ No □ Not charted

Positive findings? □ Yes □ No

If yes, location (check all that applies):

Scrotal □

Umbilical □

Femoral (usually only girls) □

**Testicular exam (males)** □ Yes □ No □ Not charted

Positive findings? □ Yes □ No

If yes (check all that applies),

Scrotal swelling/erythema/firm/tender □

Mass □

Hydrocele □

Hernia □

**Pelvic exam (females)** □ Yes □ No □ Not charted

Positive findings? □ Yes □ No

If yes (check all that applies):

Cervical Motion Tenderness □ Yes □ No □ Not charted

Blood □ Yes □ No □ Not charted

Mass □ Yes □ No □ Not charted

If yes (check all that applies):

Cervical □

Uterine □

Adnexal □

Vulvar □

Lymphadenopathy □ Yes □ No □ Not charted

Discharge □ Yes □ No □ Not charted

Vulvar Lesions □ Yes □ No □ Not charted

Erythema □ Yes □ No □ Not charted

**Rectal exam**  □ Yes □ No □ Not charted

Positive findings? □ Yes □ No

If yes (check all that applies):

Fecal Mass □

Blood □

Hemorrhoids □

**INVESTIGATIONS COMPLETED IN THE EMERGENCY DEPARTMENT**

**Blood workup:** □ Yes (complete section bellow) □ No (skip to urine)

Time of drawing blood or inserting IV (found in ED nursing notes) ____(hh):___:(mm)

Complete blood count: White blood cell(WBC)______ , Red blood cell (RBC)______,

Hemoglobin (HB) _____, Neutrophil _______, lymphocyte ________

Bands cell ______, platelet ______, MCV_______

□ Electrolytes: Sodium (Na)_____, Potassium (K)_____,

Chloride (Cl) _____, Blood urea nitrogen (BUN) _____, Creatinine ______

□ lactate_____

□ Blood Gas : PH _____, PCO2 ______, HCO3 ______, Base excess _______

□ C-reactive protein (CRP)_______

□ Blood culture: □ Specification_____________________

□ Gram stain _____________________

**IMAGING STUDIES:**

**Abdo X -ray (AXR):** □ Yes □ No

Time of AXR (found on report)_____(hh):_____:(mm)

If yes, terms found in radiologist report?

□ Appendicolith

□ Fecal loading

□ Mural thickening

□ Cecal gas

□ Small bowel dilatation

□ Air fluid levels

NOTES (please enter the summary of the report): _____________________________________________________________________________________________________________________________________________________________________________________________________________________________________________

**Ultrasound**

Fax time (on the ED Ultrasound Requisition) _______

Ultrasound completion time (on the ultrasound report) _______

Ultrasound preliminary report time _______

*If yes, terms found in radiologist report (check all that apply)?*

□ Dilated appendix

□ Aperistaltic

□ non-compressible

□ hyperechoic appendicolith with posterior acoustic shadowing

□ distinct appendiceal wall layers

□ wall thickening

□ loss of wall stratification with necrotic stages

□ echogenic prominent pericaecal and periappendiceal fat

□ periappendiceal hyperechoic structure

□ periappendiceal fluid collection

□ target appearance (axial section)

□ peri appendiceal reactive nodal prominence/enlargement (mesenteric lymphadenitis)

NOTES (enter summary of the report, include size of appendix in millimeters if visualized, e.g. 7 mm):

**CT**

Fax time _______

CT completion time_______

CT preliminary report time_______

NOTES (enter summary of the CT report):

**Consult (look for consult notes)** □ Yes □ No

Service consulted by ED: _____________________

Time of consult (written on ED chart or in surgical consult note): ___(hh)____(mm)

Any other service consulted □ Yes □ No

Name of service: _____________________

**Surgical admission** □ Yes □ No

Appendectomy date (YYYY/MM/DD)_______________Time (OR REPORT) (hh:mm)____________

**MANAGEMENT IN EMERGENCY DEPARTMENT**

**Time from Triage to Intravenous Insertion:**___hh ___mm *(in hh:mm, 24:00, e.g. 1830, as documented in RN notes)*

**Intravenous fluid:** □ Yes □ No

□ Normal saline

□ 1/2 NS + D5W

□ Ringer’s lactate

Other _________________________

Bolus (usually administered over an hour, 10-20 cc/kg □ Yes □ No

Time of Bolus initiation: ___hh ___mm *(in hh:mm, 24:00, e.g. 1830, as documented in RN notes)*

Time of Bolus completion: ___hh ___mm

Reason for Bolus fluid administration: □ Not charted

Dehydration: □

Bladder filling for ultrasound: □

Other: _____________________

Total Bolus volume given (as per MD order and RN notes) _________________________

Maintenance fluid (e.g. TKVO) □ Yes □ No

**Intravenous Antibiotics:** □ Yes □ No

If yes (check all that applies):

Ceftriaxone □

Metronidazole (Flagyl) □

Ampicillin □

Other _________________________

**Analgesia** □ Yes □ No

If yes (check all that applies):

Type of analgesia administered

Acetaminophen □

Ibuprofen □

Ketorolac □

Morphine □

Fentanyl □

Other _________________________

Route of administration (Branched logic on redcap for each medication)

PO □

IV □

Intranasal □

Dose of analgesia _____ □ milligrams □ micrograms

Time administered (hh:mm, 24:00, e.g. 1845) ___hh____mm

**Anti-emetics**

Antihistamines □

(e.g. Gravol (diphenhydrinate), Benadryl (diphenhydramine))

Ondansetron (Zofran) □

**DISPOSITION:**

□ Discharge Home □ Admission

**FOLLOW UP PLANS:**

□ Scheduled reassessment 24-48 hrs with surgery or ED

□ Pediatric Rapid Access Clinic (PRAC)

□ Family MD □ Outpatient Pediatrician

**Discharge diagnosis from ED:** __________________________________

**Service Admitted:** □ Pediatric Surgery □ General Pediatrics

□ Other : _________________

**Length of admission in days (in Meditech): ­­­­­­_________________**

**Diagnosis on discharge from admission:** _________________

**Appendectomy completed (Operating room NOTE):** □ Yes □ No

**Pathology report of appendectomy (check all that applies):**

Appendix normal □ Ulcerated □

Appendix inflamed □ Hemorrhagic □ Necrotic □ Hyperemic □

Pus filled □ Abscess formation □

Perforated □ Fecalith present □

NOTE (enter summary of Pathology report):

**Date and Time of discharge from the emergency department:**

_______/_______/_________   _____:_____ (24:00 e.g. 18:52)

**YYYY MM DD hh : mm**

**Attending ED staff who completed initial assessment:** --------------------------------------------------------

**This section completed by (Initials only)**: _____________________________

**Signature:** _______________________________ Date: _____ / _______ / _________

yyyy mm dd

**This section reviewed by (Initials only)**: _____________________________

**Signature:** _______________________________ Date: _____ / _______ / _________

yyyy mm dd

Note:_______________________________________________________________________________________________________________________________________________________________________________________________________________________________________
