# Supplementary material for: An Advanced Nursing Directive for Children With Suspected Appendicitis: Protocol for a Quality Improvement Feasibility Study
Source: JMIR Res Protoc. 2021 Oct 20;10(10):e33158. doi: 10.2196/33158 (PMC8567150; doi:10.2196/33158)
Supplement: Multimedia Appendix 4 [file resprot_v10i10e33158_app4.pdf]

Hamilton Health Sciences
RESEARCH ADMINISTRATION
New Investigator Fund

Daniela M. Bianco
Manager, Research Development & Relations
Research Administration
293 Wellington St. N., Suite 120
Hamilton, ON L8L 8E7
Email: biancdan@hhsc.ca
905 521-2100, ext. 43828
(F) 905 521-7902

June 18, 2019

Dr. Mohamed Eltorki
Pediatric
MUMC

Dear Dr. Eltorki,

Re: NIF-19463

Congratulations! We are pleased to inform you that your grant application to the Hamilton Health Sciences' New Investigator Fund (NIF) for the project entitled ***"Quality Improvement Study of An Emergency Department Advanced Nursing Directive for Appendicitis"*** has been granted final approval for funding in the amount of ***$50,000.***

You are required to set up a hospital (HHS) research account for **each new** research study that funding is obtained for. Please contact Donna Catherwood via email at catherwood@hhsc.ca or ext. 74604 to arrange a meeting with the research finance team to go over the account setup process.

You are restricted to only submitting expenditures itemized in the budget that was reviewed and approved by the NIF Scientific Peer-Review Board. Any other expenses requiring payment must be justified and incorporated into a revised budget without affecting the initial amount granted. The revised budget needs to be approved by the office of Research Administration – biancdan@hhsc.ca.

Funding for knowledge dissemination; travel are not considered eligible expenses, at this point in time; therefore, have been deducted from all budgets. The review board agreed that such requests should come back to the board and will be contingent upon proof of project finalization and evidence of acceptance for presentation/publication.

Please know that any study involving human or animal subjects requires approval by the applicable Research Ethics Board in order to claim your NIF. As well, HHS has adopted and implemented several education and training programs/resources (SOPs, GCP, TCPS2, Chart Review Tutorial and Protecting Personal Health Information Tutorial) that are available for novice investigators to complete (as applicable to their research activities). Please consult with the office of Research Administration for access to these education and training programs/resources by contacting Daniela Bianco, Manager- Research Administration via email at biancdan@hhsc.ca

Please note that an annual progress report of your study is required. Upon completion of your study, a final report must also be submitted. Please forward your report(s) to Donna Catherwood at** catherwood@hhsc.ca**. All published work must acknowledge the support of the New Investigator Fund, Hamilton Health Sciences.

If you require any further clarification or have any concerns regarding your submission, please contact Daniela Bianco at (905) 521-2100, ext. 43828. We wish success as you venture into your independent research career!

Sincerely,

HAMILTON HEALTH SCIENCES

Michelle Ghert, MD, FRCS(C)
Orthopedic Oncologist
Associate Professor, Dept. of Surgery
McMaster University
Co-Chair, Scientific Peer-Review Board
HHS New Investigator Fund

Cc: Dr. Andrew Worster

HAMILTON HEALTH SCIENCES
New Investigator Fund
Scientific Peer-Review Board Evaluation Sheet

NIF - 19463 Novice Investigator: Yesx☐
No ☐
CANDIDATE NAME: Eltorki, Mohamed *(within first 5 years of independent investigation)*

REVIEW CRITERIA

a) Achievements and Activities of the Candidate
- Publication Activity
- Other Research Activity

b) Characteristics and Abilities of the Candidate *(from mentor's assessments)*
- Background Preparation
- Motivation/initiative
- Skill at Research
- Intellectual Ability
- Originality

c) Achievements and Activities of the Mentor
- Scientific Activity
- Supervisory Record

d) Research Training Environment
- Training Program for the Candidate
- Research Resources
- Research Team Capacity

e) Potential Impact of Research on Practice and Health Services Delivery

Applications that demonstrate scientific excellence with the highest weighted score out of 10 will be awarded grants.

PROJECT SUMMARY:Given the strong evidence suggesting that ANDs (advanced nursing directives) expedite and improve quality of patient care in the ED without increasing ED resource utilization, we will investigate the effect of a new AND on patients presenting to the ED with suspected appendicitis. This QI initiative is novel as our proposed AND will empower nurses to order advanced imaging studies (ultrasound) in patients who meet specific evidence-based criteria for suspected appendicitis, a step further than what is previously reported in the literature. It will have an impact on systems and patient level outcomes, leading to potential cost savings and improved care.
The purpose of this submission to the HHS New Investigator Fund (NIF) competition is to fund this single center QI project as a proof of concept and a first step towards a multi center project to standardize and improve the ED approach for patients with suspected appendicitis.

STRENGTHS: Dr. Eltorki has a strong publication and presentation history. His letters of support indicate his commitment to and his ability to carry out this research project. It is refreshing to see a multi-disciplinary project and his support of his staff. The before/after design is an excellent choice for this project. It is also good to see that he has a program of research planned and will follow this with a large multi-centre study.

WEAKNESS(ES): It would have improved the submission if some nurses were listed as co-investigators.

HAMILTON HEALTH SCIENCES
New Investigator Fund
Scientific Peer-Review Board Evaluation Sheet

NIF - 19463
CANDIDATE NAME: Eltorki, Mohamed

Novice Investigator: Yes☐ No ☐
(within first 5 years of independent investigation)

REVIEW CRITERIA

a) Achievements and Activities of the Candidate
- Publication Activity
- Other Research Activity

b) Characteristics and Abilities of the Candidate *(from mentor's assessments)*
- Background Preparation
- Motivation/initiative
- Skill at Research
- Intellectual Ability
- Originality

c) Achievements and Activities of the Mentor
- Scientific Activity
- Supervisory Record

d) Research Training Environment
- Training Program for the Candidate
- Research Resources
- Research Team Capacity

e) Potential Impact of Research on Practice and Health Services Delivery

Applications that demonstrate scientific excellence with the highest weighted score out of 10 will be awarded grants.

PROJECT SUMMARY:

Focus is to implement and validate an evidence-informed Advance Nursing Directive (AND) for suspected appendicitis to reduce wait times by 20% (compared to current practice) in children 3-17 who present to the ED at MCH with abdominal pain. Secondary outcomes include improved pain management, decreased length of stay, reduce costs and parental anxiety. This is a single center time series analysis methodology to assess before and after the AND. It will establish proof of concept and serve as pilot for a multi-centre study. The MCH has ~ 800 visits per year for abdominal pain that is investigated for appendicitis.Currently diagnosis is dependent on physician assessment.There is strong evidence that AND are effective. This AND will include the ordering of imaging which has not been previously reported. The two phases of the PDSA cycles are planned before and after implementation of the AND. Educational sessions, visual care map will be used to disseminate the AND and gain uptake. All patients with RLQ cough, jump or percussion and ileofossal tenderness and symptoms < 4 days are eligible. Some exclusions apply. Time to discharge is recorded electronically.Study will run for one year.

STRENGTHS:

Study builds on and leverages an established strong collaborative interprofessional environment. Potentially significant patient important outcomes. The applicant completed a survey of nurses in the ED to determine their interest in developing a appendicitis AND: the majority supported. IT has also received enthusiastic endorsement from the MCH Family Council.

WEAKNESS(ES):

Pediatric Emergency Medicine Research Enrichment Program: flat fee of $5,500: Please justify further. The proposal refers to this as a pilot study and QI study: while it is directed at improving quality of care and patient experience, it seems easily to fit into a research category.

HAMILTON HEALTH SCIENCES

New Investigator Fund – Patient Advisor for Research

Lay Summary Evaluation Form

NIF-19463
NIF Principal Investigator: Eltorki, Mohamed

Hamilton Health Sciences

VISION
Best Care for All

MISSION
To provide excellent health care for the people and communities we serve and to advance health care through education and research.

REVIEW CRITERIA

1. Readability for general public at a grade 8 level
2. Clearly ties to the New Investigator Fund purpose, priorities and Goals
3. Advances and creates new knowledge that informs patient care

---

1. Is the readability for general public (at grade 8 level)? Yes ☒ Somewhat ☐ No ☐

- Is the tone appropriate for public audience?
- Is it easy to understand? Does it make sense to you?
- Do the ideas flow together in a fashion that is easy to follow?
- Is terminology explained and used sparingly if necessary: uses "everyday" language
- Are sentences short and clear?
- Are simple terms used? Is medical terminology avoided or explained in a way that makes sense to you?
- Is there any language which disturbs you, or that you find would be sensitive to other people like yourself?

Lay Reviewer Comments:
While this lay summary is clearly written, it does seem somewhat overly detailed for a lay summary. As well, did not flow from section to section and therefore was difficult to follow.

2. Does the research support the purpose and priorities of the New Investigator Fund
Yes ☐ Somewhat ☒ No ☐

- Does this research complement HHS' health care priorities/goals:
  1) *Patients – provides an excellent patient and family experience?*
  2) *People – engages, empowers and enables HHS staff to deliver our Mission?*
  3) *Sustainability – meets the health care needs of the communities we serve (now and in the future)?*
  4) *Research, Innovation and Learning – leads for the benefit of our community and the world?*
- Does this research include a team of a variety of health care providers (eg, doctors, nurses, physiotherapists, pharmacists)?
- Does the research involve other health care areas/departments at HHS?
- Does this research add to current research work being done at HHS?
- Is this research needed in order to provide important information that can be used to do more research?
- Will this research help a junior researcher develop his/her career?

Lay Reviewer Comments:
Yes, this research does involve a variety of providers and builds on other quality improvement projects.

3. Does this research advance and create new knowledge that informs patient care?
Yes ☒ Somewhat ☐ No ☐

- Will the results of this study inform new ways to help patients?
- Is there a clear understanding of what will be done with the information this research will provide?
- Will the results of this study lead to doing other research projects?
- Do you find this research interesting?

Lay Reviewer Comments:
Yes, has potential to create new knowledge to inform patient care. Fairly clear understanding of research and what will be done. It was moderately interesting.

Overall Comments:
- Is there a clear understanding of what the research is about?
- Is there a clear understanding of the value and importance for doing this research work?
- Do you understand how this research work will benefit patients?
- Will this research add important information that is needed to do more research in this area?
- Has the research work been explained clearly that it is achievable/doable?
- Will this research help develop a junior researcher's career?

This summary was quite lengthy and detailed and I feel that it did not require this level of detail which took away from the need and/or importance of this study.

Reviewer Name: Date: May 19/19
